# Sodiophilic Ag-diamane-Modulated Polypropylene Separators for High-Performance Sodium Metal Anodes

**DOI:** 10.3390/molecules30102092

**Published:** 2025-05-08

**Authors:** Gang Zhi, Zhanwei Hu, Zhuangfei Zhang, Hui Wang, Dezhi Kong, Guozhong Xing, Dandan Wang, Zhihong Mai, Tingting Xu, Xinjian Li, Ye Wang

**Affiliations:** 1 Key Laboratory of Material Physics, Ministry of Education, School of Physics, Zhengzhou University, Zhengzhou 450052, China; zhigang@zzu.edu.cn (G.Z.); hzw15539486708@gs.zzu.edu.cn (Z.H.); zhangzf@zzu.edu.cn (Z.Z.); aphwang@zzu.edu.cn (H.W.); dezhi_kong@zzu.edu.cn (D.K.); xutt@zzu.edu.cn (T.X.); 2 Institute of Microelectronics, Chinese Academy of Sciences, Beijing 100029, China; gzxing@ime.ac.cn; 3 Hubei Jiufengshan Laboratory, Wuhan 430206, China; wangdandan@jfslab.com.cn (D.W.); maizhihong@jfslab.com.cn (Z.M.)

**Keywords:** sodium metal anodes, diamane, Ag-diamane/PP, dendrite-free, uniform deposition

## Abstract

Sodium metal is a promising anode material for sodium metal batteries (SMBs) due to its high theoretical specific capacity and low electrochemical potential. However, its practical implementation is severely limited by dendrite formation, which causes short circuits and safety issues. Here, we introduce a separator modification strategy using Ag nanoparticles decorated with two-dimensional diamane on a commercial polypropylene (PP) substrate (Ag-diamane/PP) to enhance the performance of sodium metal anodes (SMAs). The synergistic effect between the sodiophilic Ag nanoparticles and the diamane network not only accelerates Na⁺ transport through the modified separator but also reduces interfacial resistance. This dendrite-suppression effect was systematically validated using in situ optical microscopy and ex situ scanning electron microscopy. Symmetric Na||Na cells incorporating the Ag-diamane/PP separator exhibit exceptional cycling stability, maintaining more than 3800 h of operation at 2 mA cm^−2^ with a capacity of 1 mAh cm^−2^. Furthermore, a full-cell configuration with a Na_3_V_2_(PO_4_)_3_@C cathode, Ag-diamane/PP separator, and Na metal anode delivers a high reversible capacity of 94.35 mAh g^−1^ and stable cycling for 270 cycles. This work highlights the Ag-diamane/PP separator as a promising solution for advancing dendrite-free SMBs with long-term cycling stability and high energy density.

## 1. Introduction

Sodium metal anodes (SMAs) hold great promise for next-generation sodium metal batteries (SMBs) due to their ultra-high theoretical specific capacity (1166 mAh g⁻¹) and low electrochemical potential (−2.714 V vs. SHE) [1,2,3,4,5,6]. However, their practical adoption is critically hindered by uncontrollable severe dendrite formation, which leads to fast capacity decay, low Coulombic efficiency (CE), and even short circuit safety concerns [7,8,9,10,11,12,13,14]. Among the diverse strategies utilized to mitigate dendrites, separator modulation to regulate Na⁺ ion flux and stabilize the plating/stripping behavior has emerged as a promising avenue [13,15,16,17,18,19,20].

Commercial polypropylene (PP) separators, widely used in lithium-ion batteries (LIBs), are inadequate for SMBs due to their low Young’s modulus (~0.5 GPa), insufficient thermal stability, and poor sodiophilicity [21,22,23]. The inferior sodiophilicity of PP separators may lead to dendrite formation in SMBs. Combined with their low Young’s modulus, this separator is more susceptible to piercing by uncontrolled dendrites. Extensive research has explored functional separators, such as graphene composites [24], 2D mesoporous polymers [25], and NASICON-type solid electrolytes [26]. Among these functional materials, diamane, a 2D diamond material with exceptional mechanical strength, thermal conductivity, and chemical inertness, has garnered significant attention [27,28]. However, pristine diamane lacks sufficient sodiophilicity for efficient Na⁺ transport to regulate the ion flux. Previous studies in our group attempted to enhance its surface chemistry via NH₂ functionalization [27] and Zn deposition [29], achieving long-term cycling stability but requiring complex multi-step synthesis (hydrolysis and annealing) [27]. These approaches, while effective, raise concerns about scalability and processability. It is necessary and timely to explore more sodiophilic materials to functionalize the surface chemistry to modulate Na ion deposition and inhibit dendrite formation [30,31,32,33].

In this work, we propose a sodiophilic Ag nanoparticle-decorated diamane/PP separator (Ag-diamane/PP) that combines the structural advantages of diamane with the electrochemical affinity of Ag for Na⁺. This design leverages two synergistic mechanisms. Ag’s strong sodiophilicity can enhance Na⁺ adsorption and lower the nucleation energy to promote planar plating. The intrinsic mechanical robustness of the diamane stabilizes the solid electrolyte interphase (SEI) and suppresses mechanical stress-induced dendrites. The sodiophicity property of the Ag can reduce the transfer resistance and increase the diffusion coefficient of Na ions, thereby alleviating concentration polarization and achieving a uniform local current density distribution. Consequently, symmetric Na||Na cells with Ag-diamane/PP separators exhibit exceptional cycling stability (>3800 h at 2 mA cm^−2^ with 1 mAh cm^−2^) and low hysteresis voltage (~41.5 mV @ 2000 h). When paired with a Na_3_V_2_(PO_4_)_3_@C (NVP@C) cathode and Na metal anode, the full cell can deliver a reversible capacity of 94.35 mAh g^−1^ and maintain 99.4% capacity retention after 270 cycles. This work demonstrates the viability of Ag-diamane/PP separators as a promising solution for dendrite-free SMBs with long cycling performance.

## 2. Results

**Figure 1:**
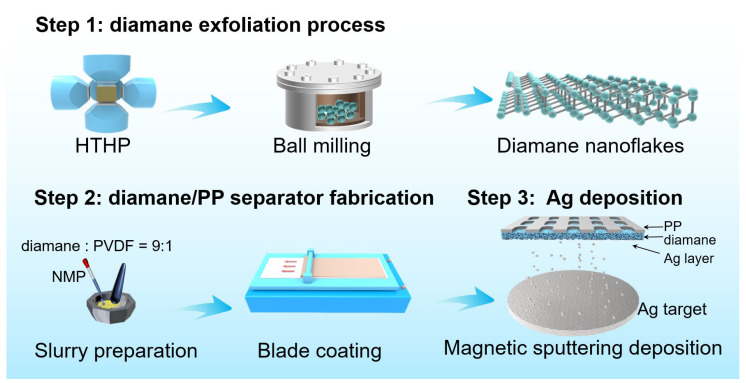
Schematic diagram of the stepwise fabrication of Ag-diamane/PP separators.

**Figure 2:**
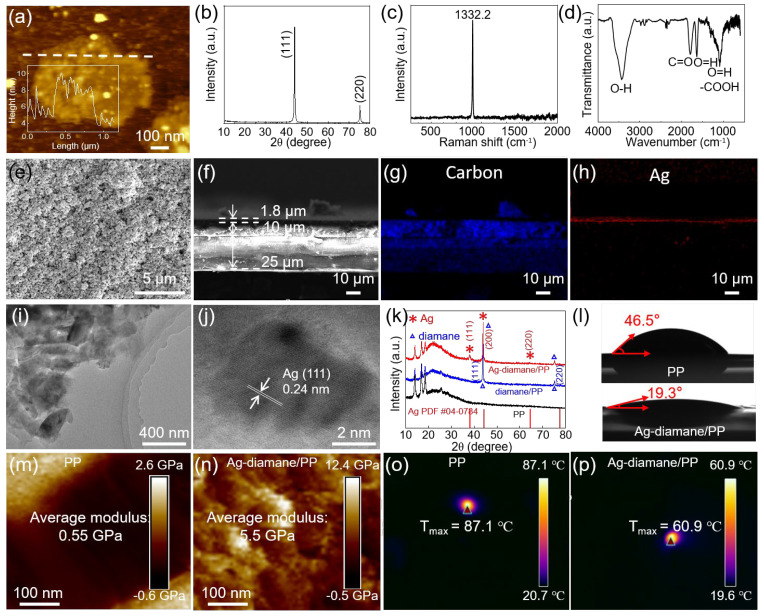
Characterization of diamane nanoflakes and Ag-diamane/PP separators. (**a**) AFM image of a diamane nanoflake. The insert is the thickness profile of the diamane nanoflake along the dashed line. (**b**) XRD, (**c**) Raman, and (**d**) FTIR spectrum of the diamane nanoflakes. (**e**) Top-view, (**f**) cross-sectional SEM images of the Ag-diamane/PP separator, and related elemental distribution of (**g**) carbon and (**h**) Ag. (**i**) TEM and (**j**) HRTEM images of the Ag-diamane nanoflakes. (**k**) XRD patterns of PP, diamane/PP and Ag-diamane/PP separators. (**l**) Contact angle measurement of the organic electrolyte on the PP (up) and Ag-diamane/PP (down) separators. Young’s modulus of (**m**) PP and (**n**) Ag-diamane/PP separators. The temperature distribution of (**o**) PP and (**p**) Ag-diamane/PP separators.

The fabrication process of the Ag-diamane/PP separator can be divided into three steps: the diamond synthesis and diamane exfoliation process, coating diamane onto the PP membrane for diamane/PP separator fabrication, and Ag deposition to form the Ag-diamane/PP separator (Figure 1). In the first step, the diamane crystals were synthesized by a high-temperature, high-pressure (HTHP) process. The diamane nanoflakes were mechanically exfoliated by a ball-milling process, followed by an acid-washing process to select the diamane nanoflakes. The synthesized diamane nanoflakes exhibited a well-defined 2D morphology, with a lateral size of ~450 nm and a thickness of approximately 4 nm (Figure 2a). Transmission electron microscopy (TEM) and high-resolution TEM (HRTEM) revealed a crystal lattice spacing of 0.21 nm, which is consistent with the (111) crystal plane of the diamond (Figure S1). X-ray diffraction (XRD) analysis confirmed that diamane belongs to the diamond phase, with distinct peaks at 43.9° and 75.3° corresponding to the (111) and (220) crystal planes of diamond (JCPDS card No. 06-0675), respectively (Figure 2b) [34]. Raman spectroscopy displays a sharp peak at 1332.2 cm^−1^, which is characteristic of the sp^3^ bonding of diamond. It is worth mentioning that there is no detectable peak associated with the sp^2^ signal orientating from the graphene, confirming the phase purity of diamane (Figure 2c) [35,36]. The surface functional groups of diamane were characterized by Fourier transform infrared spectroscopy (FITR) (Figure 2d). The peaks at 3441, 1592, 1368, and 1282 cm^−1^ were due to the O-H bond [37]. The peaks at 1869 and 1142 cm^−1^ were due to the C=O and -COOH functional groups, respectively [37,38]. These functional groups were introduced during the repeated acid treatment process. In the second step, in order to improve the affinity between diamane and the PP layer, the PP separator was treated with plasma for 300 s, followed by coating the prepared diamane nanoflakes on the prepared PP separator (pore dimensions: ~1000 nm length, ~100 nm width) by a conventional blade-coating process (Figure S2). After heating, an approximately uniform 10 μm thick diamane layer was formed on one side of the PP separator (25 μm) to form the diamane/PP separator (Figure S3). In the third step, Ag nanoparticles were deposited onto the diamane/PP separator via magnetron sputtering to produce the final Ag-diamane/PP separator. The morphology of the Ag-diamane/PP separator was similar to that of the diamane/PP separator (Figure 2e). The cross-sectional scanning electron microscopy (SEM) image and related energy-dispersive X-ray spectroscopy (EDS) elemental mapping showed a three-layer structure of a 25 μm PP layer, a 10 μm diamane layer, and 1.8 μm Ag layer (Figure 2f–h). TEM and HRTEM images confirmed that Ag nanoparticles with an average diameter of ~5 nm are uniformly dispersed on diamane, with lattice spacing of 0.24 nm (Ag (111) crystal plane) (Figure 2i,j). The XRD pattern of Ag-diamane/PP shows peaks at 43.9° and 75.3° originating from diamane, and the peaks located at 38.1°, 44.2°, 64.6° can be indexed to the (111), (200), and (220) crystal planes of Ag (JCPDS card No. 04-0784) (Figure 2k) [39,40,41].

The prepared Ag-diamane/PP separators exhibit critical properties that are suitable for sodium metal anodes, including enhanced electrolyte wettability, exceptional mechanical rigidity, and superior thermal stability with fast thermal conductivity, all of which synergistically improve interfacial kinetics and suppress dendrite growth [24]. Contact angle measurements show that the contact angle between the electrolyte and Ag-diamane/PP separator is only 19.3°, which is significantly lower than that of pristine PP (46.5°) and slightly lower than diamane/PP (19.8°) (Figure 2l and Figure S4). Notably, the Ag-diamane/PP separator achieves complete electrolyte wetting within 5 s (Figure S5). This rapid wetting behavior, attributed to hydrophilic functional groups on the surface of diamane, facilitates a fast Na^+^ transportation rate and improves the surface kinetics [42]. Mechanical testing further demonstrates an Ag-diamane/PP’s Young’s modulus of 5.5 GPa and a 3.8% improvement over diamane/PP (5.3 GPa), and an approximately 10 times higher than that of PP (0.55 GPa) (Figure 2m,n and Figure S6). With a high Young’s modulus, the Ag-diamane/PP separator provides robust resistance against dendrite penetration [43]. The Ag-diamane/PP separator demonstrates exceptional thermal stability and rapid thermal diffusion capabilities, which are critical for mitigating thermal runaway. At 100 °C, the PP separator undergoes severe deformation (Figure S7a). In comparison, both diamane/PP and Ag-diamane/PP separators retain structural integrity up to 140 °C (Figure S7b,c). To further evaluate the thermal diffusion ability of the diamane coating, localized laser heating was applied to each separator for 10 s (Figure S8). The PP separator reached a peak temperature of 87.1 °C (Figure 2o). In contrast, the center temperatures of the diamane/PP and Ag-diamane/PP separators are reduced to maximum temperatures of 66.4 °C and 60.9 °C, respectively (Figure S9 and Figure 2p).

**Figure 3:**
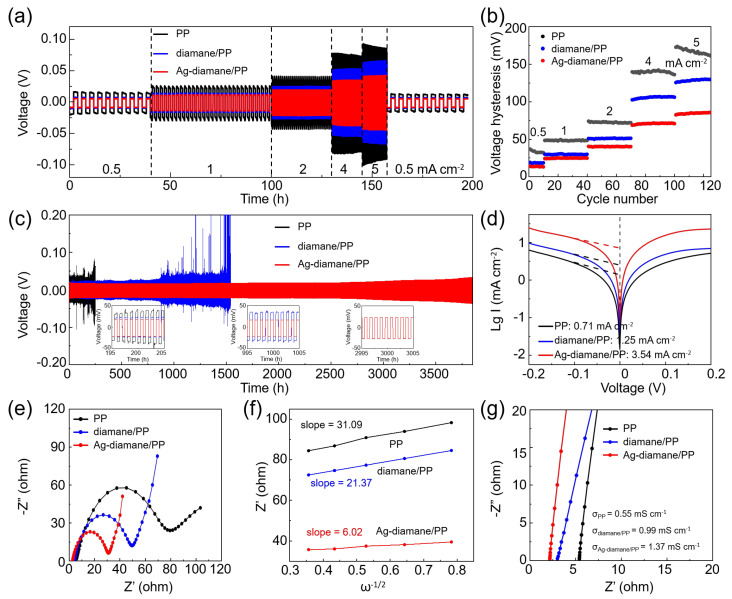
Electrochemical performance of symmetric cells employing PP, diamane/PP, and Ag-diamane/PP separators. (**a**) The rate of capability at current densities of 0.5, 1, 2, 4, and 5 mA cm^−2^ and (**b**) related voltage hysteresis. (**c**) Long-term cycling at 2 mA cm^−2^ with 1 mAh cm^−2^. The insets are the enlarged voltage profiles of the cells with various separators at ~200, ~1000, and ~3000 h. (**d**) Tafel curves, (**e**) EIS spectra, (**f**) derived diffusion coefficient, and (**g**) ionic conductivity.

To assess the impact of the Ag-diamane/PP separator on Na metal deposition reversibility, symmetric Na||Na cells with PP, diamane/PP, and Ag-diamane/PP were cycled at varying current densities (0.5–5 mA cm^−2^). As the current density gradually increased from 0.5 to 5 mA cm^−2^ (Figure 3,b), the cell employing the Ag-diamane/PP separator exhibited minimal voltage hysteresis (for example, 13.6 mV at 0.5 mA cm^−2^, and 84.8 mV at 5 mA cm^−2^) compared to cells with PP and diamane/PP separators (Figure 3b and Table S1). The flat voltage profiles for the cell with the Ag-diamane/PP separator indicate efficient Na^+^ diffusion kinetics with the assistance of the diamane layer and Ag nanoparticles. Long-term cycling performance was further measured at 2 mA cm^−2^ with 1 mAh cm^−2^. As shown in Figure 3c, the cell employing the PP separator exhibited unstable voltage profiles, as evidenced by an increased hysteresis voltage from 75.83 mV at 150 h to 173.6 mV at 250 h. The cell rapidly failed at 253 h, which may have been due to the dendritic penetration [19,44,45]. In contrast, the cell employing the diamane/PP separator displayed moderate stability up to 830 h with a small hysteresis voltage of 57.7 mV. However, the voltage profile became unstable until it short-circuited after 1540 h. Remarkably, the cell employing the Ag-diamane/PP separator sustains stable operation until 3800 h with a small polarization shift (41.5 mV at 2000 h, 52.3 mV at 3000 h) at 2 mA cm^−2^ with 1 mAh cm^−2^ (Figure 3c). It is worth mentioning that the cell with the Ag-diamane/PP separator also exhibits excellent long cycling at high current density with a large areal capacity. For example, it shows a long cycle lifespan of more than 1000 h at a high current density of 5 mA cm^−2^ and a large areal capacity of 10 mAh cm^−2^ (Figure S10). This performance enhancement arises from the dual functionality of the Ag-diamane layer. The diamane layer can effectively suppress dendrite propagation via its high Young’s modulus property. The diamane and Ag nanoparticles homogenize Na^+^ flux through sodiophilic interactions, thereby achieving long cycling performance [31].

The diffusion kinetics of Na⁺ with the prepared Ag-diamane/PP separator were evaluated through the exchange current density analysis, the charge transfer resistance measurements, and diffusion coefficient calculations. As shown in Figure 3d, Tafel polarization curves revealed that the cell with the Ag-diamane/PP separator exhibits the highest exchange current density of 3.54 mA cm^−2^, which is much higher than that of the PP separator (0.71 mA cm^−2^) and diamane/PP separators (1.25 mA cm^−2^). This result confirms accelerated charge-transfer kinetics at the Na anode interface enabled by the Ag-diamane layer [46,47,48]. The measurement of electrochemical impedance spectroscopy (EIS) further quantified the interfacial dynamics, demonstrating a dramatically reduced charge transfer resistance of 27.3 Ω compared to the PP separator (76.8 Ω) and diamane/PP separator (47.5 Ω) (Figure 3e and Table S2). The Na^+^ diffusion coefficient (D_Na+_) is derived from the Warburg region of the EIS spectra, showing a linear dependence of the real impedance component Z_re_ on the inverse square root of the angular frequency (ω^−1/2^) [44,49,50]. The Na⁺ diffusion coefficient for cells with Ag-diamane/PP separators is 6.02 × 10^−13^ m^2^ s^−1^, which is higher than that of PP (2.26 × 10^−14^ m^2^ s^−1^) and diamane/PP separators (4.78 × 10^−14^ m^2^ s^−1^) (Figure 3f). This value is also higher than that of PP/NH_2_-diamane separators [27]. Moreover, the Na^+^ ionic conductivity is also calculated according to σ = D/(SR), where D, S, and R are the thickness, the size of the separator, and the electrolyte resistance derived from EIS [29]. The ionic conductivity of the Ag-diamane/PP is 1.37 mS cm^−1^, which is superior to that of diamane/PP (0.99 mS cm^−1^) and PP separators (0.55 mS cm^−1^), as shown in Figure 3g and Table S2. These results show that the Ag-diamane/PP separator can effectively mitigate interfacial impedance and enhance ion transport, which is critical for stabilizing the high-rate and long cycling Na plating/stripping process [51,52,53].

**Figure 4:**
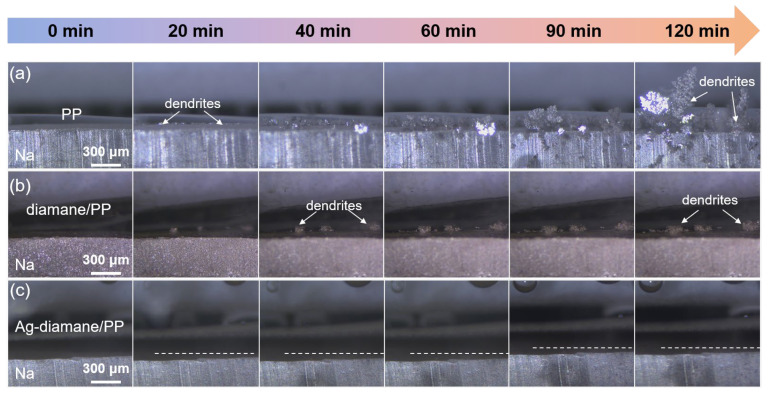
In situ optical microscopy of Na deposition morphology in cells with (**a**) PP, (**b**) diamane/PP, and (**c**) Ag-diamane/PP separators at 4 mA cm^−2^ for 120 min.

To investigate the mechanism for improved electrochemical performance with the assistance of the Ag-diamane/PP separator, a systematic analysis of deposition morphology evolution and Na^+^ flux regulation was conducted. In situ optical microscopy was employed to dynamically monitor dendrite formation during Na plating at 4 mA cm^−2^ (Figure 4). Cells utilizing PP and diamane/PP separators exhibited prolific dendritic growth within 20 min, progressing to large mossy-like dendrites by 120 min (Figure 4a,b). In contrast, the cell with the Ag-diamane/PP separator maintained a planar and dendrite-free morphology throughout 120 min deposition (Figure 4c). Similar results are also observed in ex situ SEM images at the micro-meter scale. As shown in Figure S11, at a deposition capacity of 0.1 mAh cm^−2^, the deposition morphologies of the Na metals with the PP, diamane/PP, and Ag-diamane/PP separators are almost the same. However, after a deposition capacity of 4 mAh cm^−2^, the Na metal with the Ag-diamane/PP separator still maintains smooth and dendrite-free morphology, while the Na metals with PP and diamane/PP separators show dendrite morphology.

**Figure 5:**
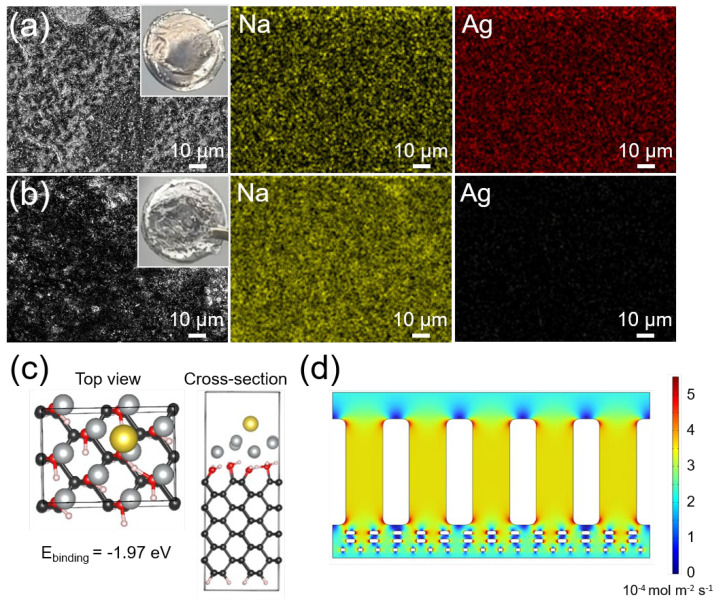
Mechanism investigation of Na metal deposition using Ag-diamane/PP separators. Optical images and elemental mappings of the (**a**) Ag-diamane/PP separator and (**b**) Na metal disassembled from Na||Na symmetric cells after 20 cycles at 1 mA cm^−2^ with 1 mAh cm^−2^. (**c**) The DFT calculation for the binding energy between Na^+^ and Ag on the diamane layer. (**d**) Simulated Na^+^ distribution through the Ag-diamane/PP separator.

To elucidate the origin of sodiophilicity, post-cycling analysis was conducted on cells subjected to 20 cycles of Na plating/stripping at 1 mA cm^−2^ with 1 mAh cm^−2^. As shown in Figure 5a,b, the EDS elemental mapping of the Ag-diamane/PP separator and Na metal anode revealed that there was no Ag on the surface of the Na electrode, whereas both Ag and Na were observed on the Ag-diamane/PP separator. Further analysis from the XRD measurement of the Ag-diamane/PP separator showed that Ag was still stable at the diamane surface (Figure S12). The density functional theory (DFT) calculation showed that the binding energy between Na ions and Ag was as high as −1.97 eV (Figure 5c), surpassing interactions with diamane (−0.81 eV) and pristine PP (−0.023 eV) [54]. This enhanced affinity facilitated Na^+^ adsorption and redistribution, enabling uniform ion flux [55]. COMSOL Multiphysics simulations further elucidated ion flux regulation. Pristine PP separators exhibit localized current density peaks at macropores, generating hot spots (Figure S13), leading to heterogeneous Na nucleation and dendritic growth. In contrast, the Ag-diamane/PP separator homogenizes ion flux (Figure 5d). This mitigation of localized charge accumulation, accompanied by the sodiophilic property of diamane and Ag, significantly suppresses dendrite formation and promotes Na dendrite-free deposition.

The low hysteresis voltage and long cycling stability of the Ag-diamane/PP separator arose from the synergistic physicochemical attributes of diamane and Ag nanoparticles. The sodiophilic nature of diamane and Ag facilitated a homogenous Na^+^ flux distribution, promoting uniform sodium nucleation and deposition. The sodiophilic property synergistically originated from diamane and Ag nanoparticles. It not only facilitated homogeneous ion distribution but also reduced the charge transfer resistance, accelerating ion transportation. Mechanically, the Ag-diamane coating enhanced the separator’s Young modulus to 5.5 GPa, providing a strong shield against dendrite penetration. Concurrently, its high thermal diffusion rate and stability ensured rapid heat dissipation during high rates of operation, circumventing thermal runaway risks. In addition, the Ag-diamane/PP separator also exhibited excellent electrolyte wettability and accelerated ion migration. With all of these advantages, the Ag-diamane/PP separator stabilized the Na metal surface, enabling sustained long cycling for more than 3800 h with minimal hysteresis voltage.

**Figure 6:**
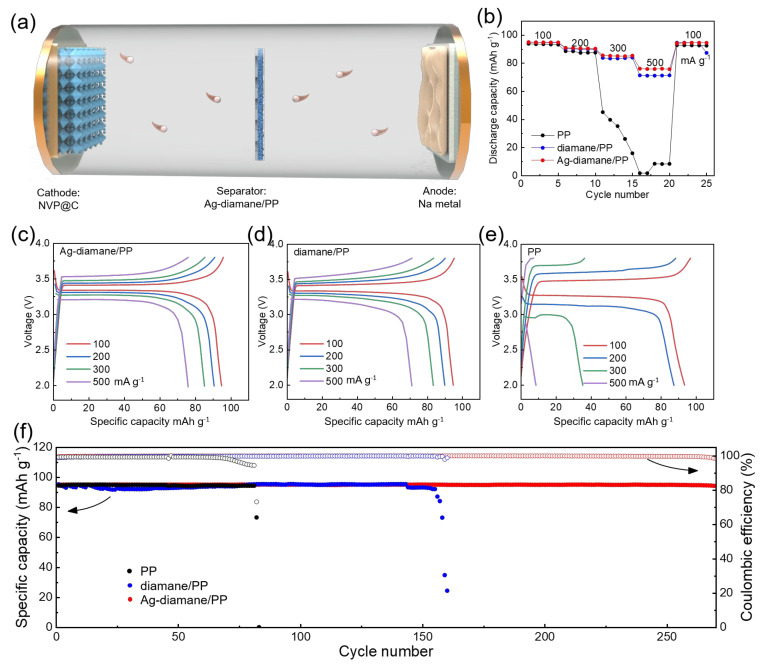
Full cell performance with PP, diamane/PP, and Ag-diamane/PP separators. (**a**) Schematic diagram of the full cell configuration using NVP@C, Ag-diamane/PP, and Na metal as the cathode, separator, and anode, respectively. (**b**) The rate capability at 100, 200, 300, and 500 mA g^−1^ corresponding galvanostatic charge–discharge (GCD) curves of (**c**) PP, (**d**) diamane, and (**e**) Ag-diamane/PP separators. (**f**) The long cycling performance of the full cells at 100 mA g^−1^ and corresponding coulombic efficiency.

To validate the practical utility of the Ag-diamane/PP separator, a full SMB was constructed using an NVP@C cathode and a sodium metal anode in conjunction with the Ag-diamane/PP separator (Figure 6a). The Ag-diamane/PP-based full cell exhibited a superior rate of capability, delivering specific capacities of 94.7, 90.6, 85.2, and 75.9 mAh g^−1^ at 100, 200, 300, and 500 mA g^−1^, respectively (Figure 6b and Table S3). These values are higher than those of PP and diamane/PP separators, especially at a high current density (for example, 500 mA g^−1^). Furthermore, the Ag-diamane/PP cell exhibits minimal voltage hysteresis at all current densities (Figure 6c–e), indicating enhanced interfacial kinetics and reduced polarization. Long-term cycling performance at 100 mA g^−1^ reveals that the full cell with the Ag-diamane/PP separator delivers a high discharge capacity of 94.35 mAh g^−1^, retaining 99.4% of its initial discharge capacity (94.9 mAh g^−1^) over 270 cycles (Figure 6f). This corresponds to a negligible decay rate of 0.002% per cycle, accompanied by a high average coulombic efficiency of 99.88% (Figure 6f and Figure S14).

## 3. Materials and Methods

### 3.1. Diamane Synthesis

Diamane nanoflakes were synthesized using a modified HTHP method combined with mechanical exfoliation, as detailed in our previous work [27]. Briefly, high-purity microdiamonds were subjected to HTHP processing to synthesize diamond crystals. These crystals were then mechanically exfoliated into thin nanoflakes using a ball-milling system followed by acid etching and subsequent centrifugal purification to obtain pristine diamane nanoflakes. The diamane/PP composite separator was fabricated via a slurry-coating technique. Diamane nanoflakes (90 wt%) and polyvinylidene fluoride (PVDF, 10 wt%) were mixed and ground for at least 10 min, and then several drops of N-methylpyrrolidone (NMP) were introduced into the mixture and agitated by a Vortex-Genie2 mixer for approximately 15 min to form a homogeneous slurry. This slurry was cast onto a Celgard 2035 PP membrane using a doctor-blade technique. Prior to the coating process, the PP separator was treated with an argon atmosphere plasma treatment at a power of 30 W for 300 s. The assembly was dried under a vacuum at 60 °C for 12 h to enhance the adhesion between the diamane and PP membrane. Lastly, Ag was deposited onto the diamane/PP separator by a magnetron sputtering process at 40 W with 100 s.

### 3.2. Characterization of Materials

The morphology and structural properties of the Ag-diamane/PP separator were systematically analyzed using SEM (JEOL, JEM-6700, Tokyo, Japan) and TEM (JEOL, JEM-2100, Tokyo, Japan). The elemental distribution of the Ag-diamane/PP separator was performed via EDS (X-Max^N^, Oxford Instruments, Oxfordshire, UK) on the SEM instrument. The crystallographic phases of diamane and Ag were characterized by XRD using Cu-Kα radiation (*λ* = 1.5406 Å) at an operating voltage of 40 kV (Rigaku SmartLab SE diffractometer, Rigaku, Tokyo, Japan). Surface chemical states and bonding configurations were probed using FTIR (Bruker VERTEX 70V, Rosenheim, Germany). Raman spectra tests were carried out using a confocal Raman spectrometer (Horiba, Labram HR Evolution, Horiba, Kyoto, Japan). The temperature distribution of the separator was measured by an infrared camera when a laser beam shone onto their surface at a power of 400 mW for 10 s. In situ optical microscopy (Cewei, LW750LJT, Shanghai, China) was employed to dynamically monitor Na deposition behavior. Symmetric Na||Na cells were assembled in an argon-filled glovebox (<0.1 ppm H_2_O/O_2_) using two Na foil electrodes and the Ag-diamane/PP separator, with 300 μL of electrolyte (1 M NaPF_6_ in diglyme). A constant current density of 4 mA cm^−2^ was applied during real-time imaging to investigate the Na deposition.

### 3.3. COMSOL Simulation

The Na^+^ flux distribution across the Ag-diamane/PP separator was simulated using COMSOL Multiphysics (version 6.0). A two-dimensional geometric model of the cell with dimensions of 4 μm × 2 μm was constructed. The size of the diamane nanoflakes was set to 160 × 80 nm, and the size of the Ag nanoparticles was set to 80 × 60 nm. The electrolyte was 1 M NaPF_6_ in diglyme. The ion distribution was governed by the Nernst–Planck equation for neutral conditions. The interfacial kinetic behavior of the separator was modeled using the linear Butler–Volmer kinetic equation.

### 3.4. Electrochemical Performance Evaluation

All cells were fabricated in an Ar-filled glovebox (<0.1 ppm H_2_O and O_2_). The symmetrical Na||Na cells were configured with a CR2032 coin cell configuration, consisting of two Na foil electrodes, a separator (PP, diamane/PP, and Ag-diamane/PP), and 70 μL of electrolyte (1 M NaPF_6_ in diglyme). The electrochemical performance of these cells with the various separators was measured by a Neware charging/discharging battery tester at ambient temperature. EIS measurement was carried out with an electrochemical workstation (Biologic VMP-3 workstation, Seyssinet-Pariset, France) across a frequency range of 10^5^–0.01 Hz with an AC amplitude of 10 mV.

For the full-cell evaluation, a Na metal anode, an Ag-diamane/PP separator, and an NVP@C cathode were integrated into a CR2032 configuration filled with 70 μL of electrolyte. Details of the fabrication process of the NVP@C cathode can be found in our previous study [47]. The mass of the NVP@C was approximately ~2 mg cm^−2^. The rate capability of the full cell was evaluated at different current densities ranging from 100 to 500 mA g^−1^. The long-term cycling performance of the full cell was measured at 100 mA g^−1^ within a potential window of 2.0–3.8 V. The current density for the full cell was calculated based on the mass of the cathode material.

## 4. Conclusions

In summary, an Ag-diamane/PP separator was designed and fabricated to homogenize sodium ion flux and regulate deposition behavior for sodium metal anodes. With this design, the Ag-diamane/PP separator significantly enhanced interfacial kinetics, achieving a high Na⁺ diffusion coefficient of 6.02 × 10^−13^ m^2^ s^−1^ and a reduced charge transfer resistance of 27.3 Ω. These properties enable a symmetric Na||Na cell to sustain stable cycling for 3800 h at 2 mA cm^−2^ with 1 mAh cm^−2^ and minimal polarization (41.5 mV at 2000 h). The dendrite-suppressing capability stems from the separator’s advantages, including sodiophilic diamane nanoflakes and Ag nanoparticles, the mechanically robust diamane layer, as well as the fast thermal diffusion ability and good electrolyte wettability. In full-cell configurations with an NVP@C cathode, the Ag-diamane/PP separator delivers a high reversible capacity of 94.35 mAh g^−1^ at 100 mA g^−1^, retaining 99.4% capacity over 270 cycles with minimal decay of 0.002% per cycle and a high coulombic efficiency of 99.88%.

## Data Availability

The data presented in this study are available upon request from the corresponding author.

## Supplementary Materials

The following supporting information can be downloaded at https://www.mdpi.com/article/10.3390/molecules30102092/s1, Figure S1: (a) TEM and (b) HRTEM images of diamane nanoflakes. Figure S2: SEM images of PP separator. Figure S3: Cross-sectional SEM image of diamane/PP separator and corresponding EDS carbon elemental mapping. Figure S4: Contact angle between the organic electrolyte and the diamane/PP separator. Figure S5: The wetting behavior of the PP, diamane/PP, and Ag-diamane/PP separators using 1 M NaPF6 in diglyme organic electrolyte. Figure S6: Young’s modulus of diamane/PP separator. Figure S7: Thermal shrinkage images of the PP, diamane/PP, and Ag-diamane/PP separators at various temperatures. Figure S8: Schematic illustration of the setup of the infrared thermography test. Figure S9: Temperature distribution of diamane/PP separators. Figure S10: Long-term cycling performance of the cell with the Ag-diamane/PP separator at 5 mA cm^−2^ and 10 mAh cm^−2^. Figure S11: SEM images of the Na electrode with (a) PP, (b) diamane/PP, and (c) Ag-diamane/PP separators at deposition capacities of 0.1, 2, and 4 mAh cm^−2^. Figure S12: XRD pattern of the Ag-diamane/PP separators after discharging at 0.2 mA cm^−2^ for 5 h. Figure S13: Simulated Na^+^ distribution across the PP and diamane/PP separators. Figure S14: Coulombic efficiency in the range of 95–101% for the full cell performance with PP, diamane/PP, and Ag-diamane/PP separators. Table S1: Average voltage hysteresis of symmetric Na||Na cells with PP, diamane/PP, and Ag-diamane/PP separators at various current densities. Table S2: The exchange current density, charge transfer resistance (Rct), ionic diffusion coefficient and ionic conductivity of the cells with PP, diamane/PP, and Ag-diamane/PP separators. Table S3: Comparison of the average discharge capacities and polarization voltages of full cells with PP, diamane/PP, and Ag-diamane/PP separators at various current densities.
