# Supplementary material for: Sodiophilic Ag-diamane-Modulated Polypropylene Separators for High-Performance Sodium Metal Anodes
Source: Molecules. 2025 May 8;30(10):2092. doi: 10.3390/molecules30102092 (PMC12114538; doi:10.3390/molecules30102092)
Supplement: Supplementary file 1 [file molecules-30-02092-s001.zip › molecules-3548217-supplementary.pdf]

Supporting Information

Sodiophilic Ag-diamane-Modulated Polypropylene Separators for High-Performance Sodium Metal Anodes

Gang Zhi [1], Zhanwei Hu [1], Zhuangfei Zhang [1], Hui Wang [1], Dezhi Kong [1], Guozhong Xing [2], Dandan Wang [3], Zhi-hong Mai [3], Tingting Xu [1], Xinjian Li [1], and Ye Wang [1],

[1] Key Laboratory of Material Physics, Ministry of Education, School of Physics, Zhengzhou University, Zheng-zhou 450052, China;

[2] Institute of Microelectronics, Chinese Academy of Sciences, Beijing, 100029, China;

[3] Hubei Jiufengshan Laboratory, Wuhan, Hubei Province 430206, China;

*Correspondence: e-mail: lixj@zzu.edu.cn (X.L.); wangye@zzu.edu.cn (Y.W.);

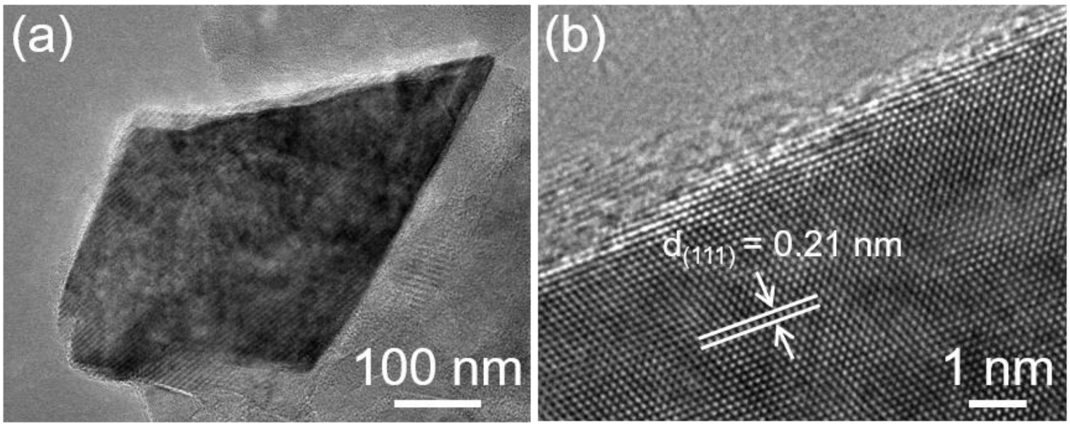

Figure S1. (a) TEM and (b) HRTEM images of diamane nanoflakes.

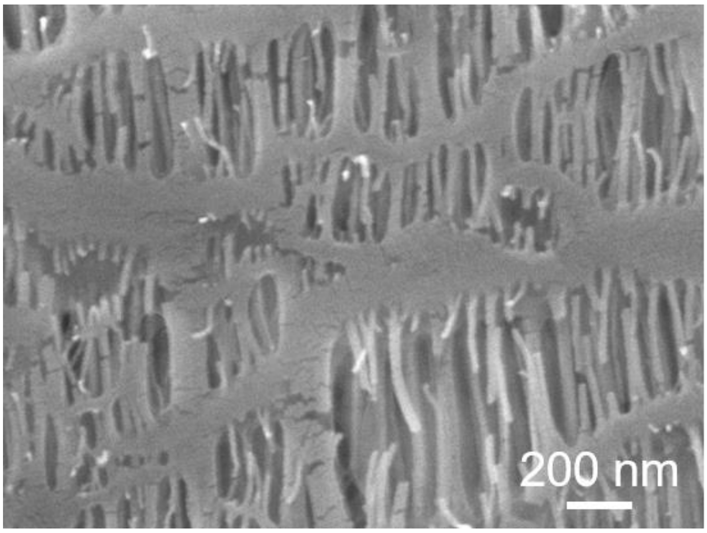

Figure S2. SEM images of PP separator.

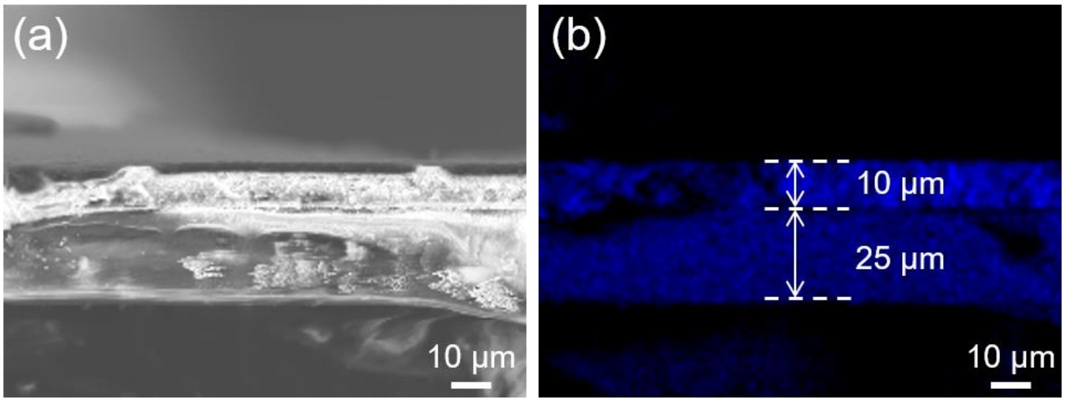

Figure S3. (a) Cross-sectional SEM image of diamane/PP separator, and (b) corresponding EDS carbon elemental mapping.

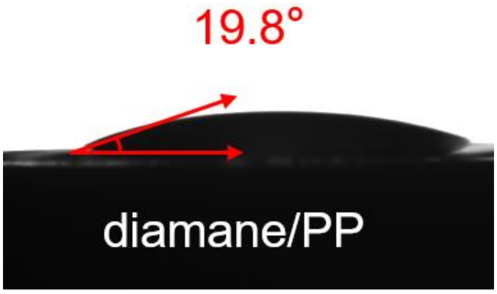

Figure S4. Contact angle between the organic electrolyte and the diamane/PP separator.

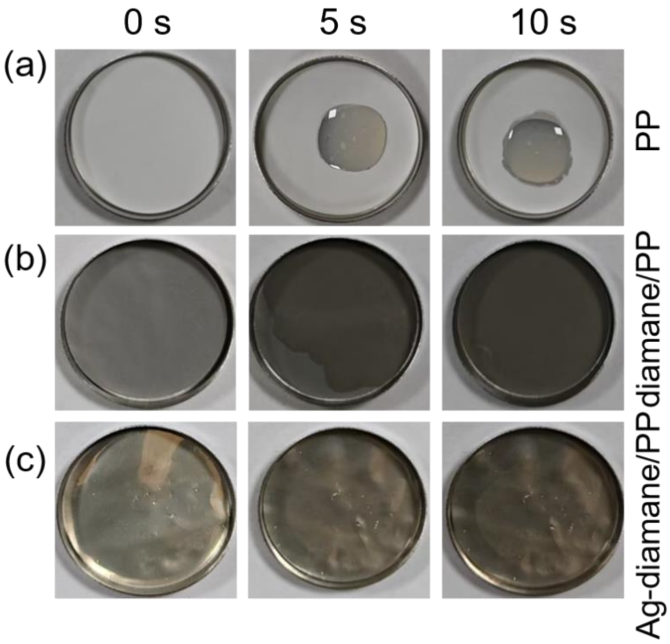

Figure S5. The wetting behavior of the PP, diamane/PP and Ag-diamane/PP separators using 1 M $NaPF_6$ in diglyme organic electrolyte.

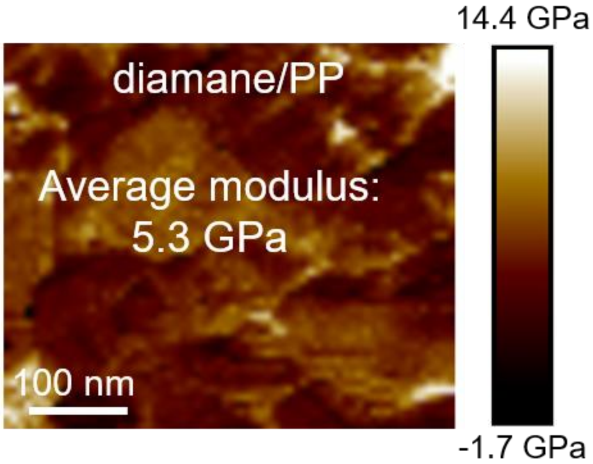

Figure S6. Young's modulus of diamane/PP separator.

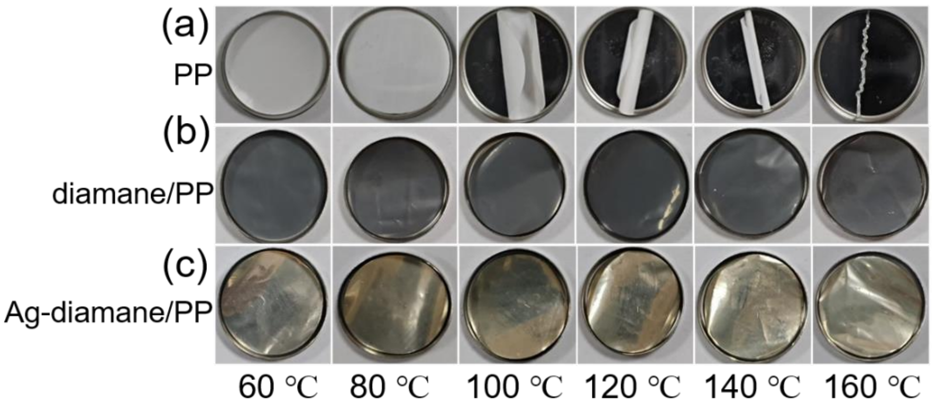

Figure S7. Thermal shrinkage images of the PP, diamane/PP, and Ag-diamane/PP separators at various temperatures.

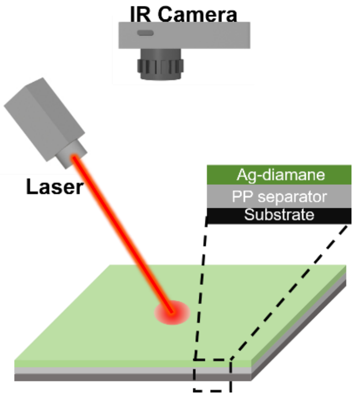

Figure S8. Schematic illustration of the setup of the infrared thermography test.

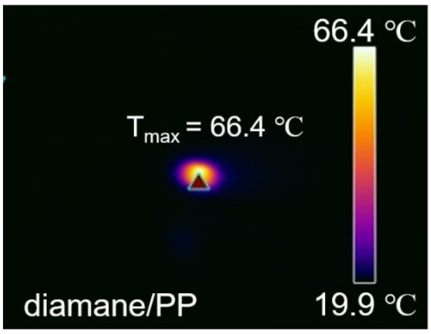

Figure S9. Temperature distribution of diamane/PP separators when a laser beam is shone on its surface with a power of 400 mW for 10 s.

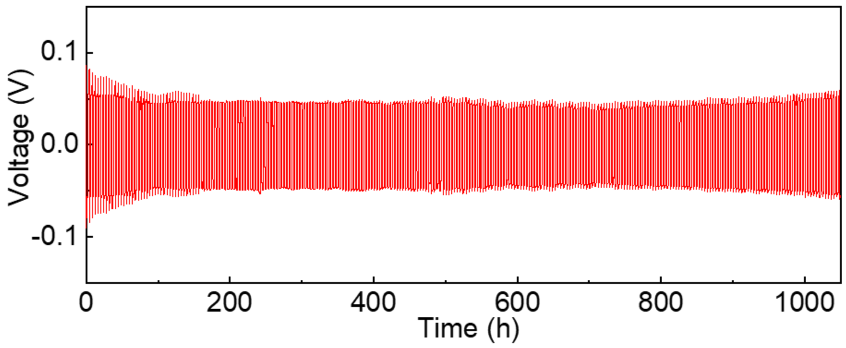

Figure S10. Long-term cycling performance of the cell with Ag-diamane/PP separator at 5 mA cm^{-2} with 10 mAh cm^{-2}.

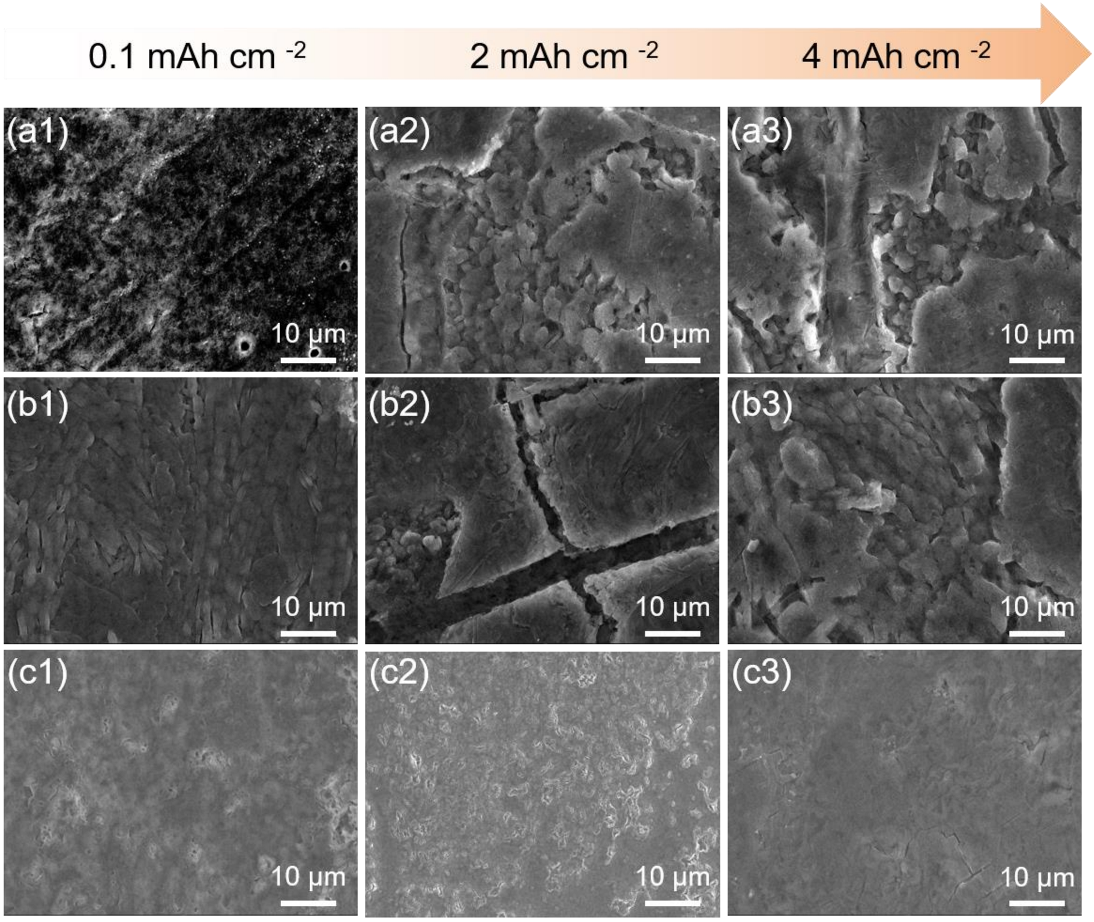

Figure S11. SEM images of the Na electrode with (a) PP, (b) diamane/PP, and (c) Ag-diamane/PP separators at deposition capacities of 0.1, 2, and 4 mAh cm^{-2}.

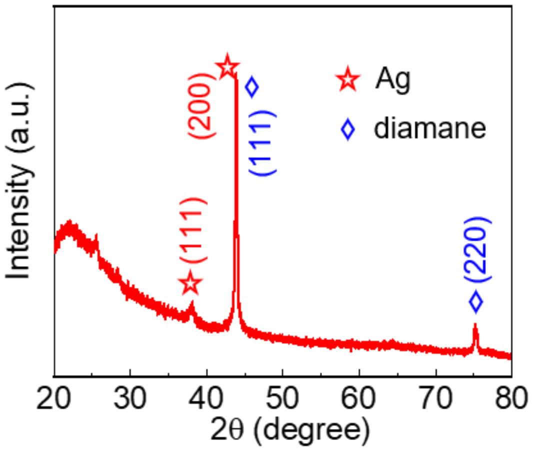

Figure S12. XRD pattern of the Ag-diamane/PP separators after discharging at 0.2 mA cm^{-2} for 5 h.

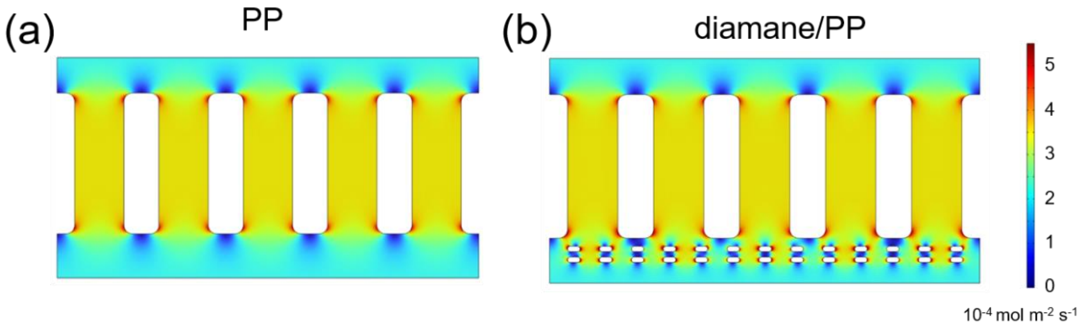

Figure S13. Simulated Na^+ distribution across the (a) PP and (b) diamane/PP separators.

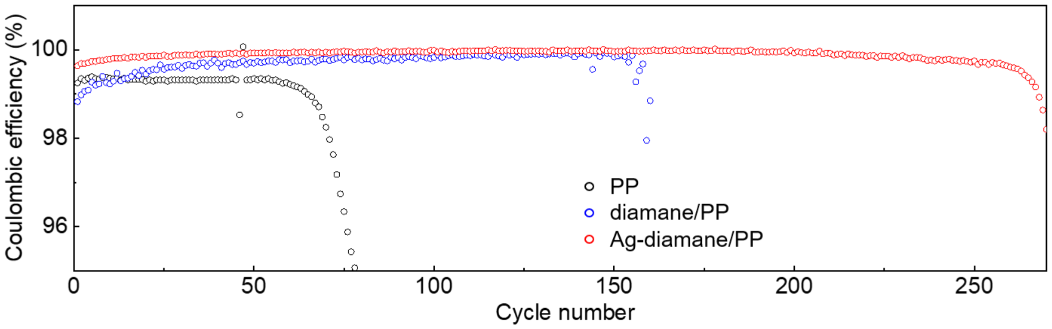

Figure S14. Coulombic efficiency in the range of 95-101% of the full cell performance with PP, diamane/PP and Ag-diamane/PP separators.

Table S1. Average voltage hysteresis of symmetric Na||Na cells with PP, diamane/PP, and Ag-diamane/PP separators at various current densities.

Separator	0.5 mA cm^{-2}	1 mA cm^{-2}	2 mA cm^{-2}	4 mA cm^{-2}	5 mA cm^{-2}
PP	33.5	48.4	72.5	139.7	165.9
diamane/PP	18.5	29.8	51.0	105.7	128.7
Ag-diamane/PP	13.6	24.8	40.2	70.8	84.8

Table S2. The exchange current density, charge transfer resistance (R_{ct}), ionic diffusion coefficient and ionic conductivity of the cells with PP, diamane/PP, and Ag-diamane/PP separators.

Separator	Exchange current density (mA cm^{-2})	R_{ct} (Ω)	Ionic diffusion coefficient (m^2 s^{-1})	Ionic conductivity (mS cm^{-1})
PP	0.71	86.8	2.26×10^{-14}	0.55
diamane/PP	1.25	47.5	4.78×10^{-14}	0.99
Ag-diamane/PP	3.54	27.3	6.02×10^{-13}	1.37

Table S3. Comparison of the average discharge capacities and polarization voltages of full cells with PP, diamane/PP, and Ag-diamane/PP separators at various current densities.

Current density ($mA\ g^{-1}$)	Average discharge capacity ($mAh\ g^{-1}$)			Polarization voltages (V)		
	PP	diamane/PP	Ag-diamane/PP	PP	diamane/PP	Ag-diamane/PP
100	93.4	94.6	94.7	0.17	0.14	0.08
200	87.9	90.1	90.6	0.48	0.22	0.14
300	32.5	83.6	85.2	0.70	0.31	0.20
500	5.8	71.2	75.9	1.03	0.43	0.34
